# Frontal Sinus Fractures: An Evaluation of Injury Parameters and Operative Variables on Surgical Outcomes

**DOI:** 10.3390/cmtr19010001

**Published:** 2025-12-23

**Authors:** George Cove, Declan Hughes, Christopher Zerafa, Simon Holmes

**Affiliations:** 1 Department of Oral and Maxillofacial Surgery, Royal London Hospital, Barts Health Trust, London E1 1FR, UK; 2 Barts and the London School of Medicine and Dentistry, Queen Mary University of London, Mile End, London E1 4NS, UK

**Keywords:** maxillofacial trauma, frontal sinus, skullbase, frontonasal outflow duct, post-operative complications, cerebrospinal fluid leakage, infection, cranialisation

## Abstract

Background: Frontal sinus (FS) injuries carry high morbidity; however, currently, there is no universally agreed-upon treatment approach for frontal sinus and frontobasal trauma. Objective: This study sets out to evaluate surgical outcomes in frontal reconstruction, looking at how fracture patterns and operative variables impact complication rates. Methods: This was a retrospective cross-sectional study which identified a cohort of 137 patients between the years 2015 and 2022 who sustained frontal sinus fractures at a level one major trauma centre in Central London. The electronic patient record (EPR) and pre-operative computed tomography (CT) were analysed to assess the following factors: patient demographics, injury parameters, surgical technique, and complications. Statistical tests included Pearson’s chi square for categorical variables/nominal data. Mann–Whitney U and Kruskal–Wallis H tests were also used to analyse continuous variables. Results: Overall, 12 of the 91 patients who were treated surgically had major complications (*n* = 12, 13.2%). In total, 5.5% (*n* = 5) had return to theatre (RTT) for cerebrospinal fluid (CSF) leaks, 5.5% for infection and 2.2% (*n* = 2) for haematoma or bleeding. FS fracture complexity was predictive of RTT (*p* = 0.015) and CSF leak (*p* = 0.015). Frontobasal complexity was predictive of post-operative infection (*p* = 0.047). Neurosurgical operative involvement and cranialisation was predictive of post-operative infection, CSF leak, and RTT. Conclusions: Understanding risk profiles in the management of FS fractures is vital in order to help clinicians mitigate these risks and also to better educate patients, including during the consent process. Further research could look at the medical and social risk factors that increase complication rates in this patient cohort.

## 1. Introduction

Frontal sinus (FS) fractures account for 5 to 15% of all facial fractures and are most often the result of high-velocity craniofacial trauma, such as motor vehicle collisions, assault, or falls [1,2]. These injuries are clinically significant, not only for their impact on facial aesthetics, but also for their potential to disrupt the underlying cranial structures, often necessitating combined neurosurgical and maxillofacial input. Approximately 25% of FS fractures solely involve the anterior table [3]; however, concomitant anterior and posterior table fractures are present in the majority of cases (57–62%) [4,5]. Isolated posterior table FS fractures are much rarer and occur in 1–7% of cases [4]. Involvement of the posterior table FS introduces a retrograde pathway for environmental contaminants to enter the intracranial space. Consequently, there is a risk of serious complications, such as meningitis, cerebral abscess, and cerebrospinal fluid (CSF) leakage [4]. Furthermore, the frontonasal outflow duct (FNOD) is disrupted in 13–55% of FS injuries [6]. Failure to identify FNOD disruptions can predispose patients to an increased risk of mucocele formation as well as other complications [7].

High-impact forces can be transmitted through the frontal vault to propagate posteriorly to the anterior cranial base [8]. This fracture pattern, known as a frontobasal injury, accounts for 3–5% [9,10] of all craniomaxillofacial fractures. Fracture extension through to the cranial base increases the risk of intracerebral complications. Notably, CSF leakage occurs in 10–30% [11] of cases, whereas pneumocephalus can be seen in 10–50% of cases [12].

Management of frontal bone, frontal sinus, and anterior skull base injuries ranges from conservative measures to invasive multidisciplinary surgical management. Surgical options are presented sequentially, ranging from simple open reduction and internal fixation (ORIF) of fractured bone segments to more complex interventions, such as obliteration (elimination of sinus mucosa and occlusion of the sinus with autologous tissue) and cranialisation (removal of the posterior table and obstruction of FNOD). Minimally invasive options have also been discussed in the literature, with endoscopic approaches being utilised to minimise morbidity [13]. Finally, reconstruction of the pre-traumatised anatomy is critical to avoid obvious frontal deformity and restore harmony to the facial upper third.

Currently, there is no universally agreed-upon treatment approach for FS and frontobasal trauma, with much of the existing research originating from single-centre retrospective cohort studies involving relatively small sample sizes. The primary objective in managing the frontal vault is the aim of restoring form and function, including precise restoration of the forehead contour/bossing with special respect to the restoration of interfacing structures to facilitate an anatomically accurate reconstruction of any concomitant trauma to the orbital, zygomatic, or nasoethmoidal regions. In addition, treatment strategies aim to minimise the risk of both early and late complications, including cerebrospinal fluid leakage, infection, and mucocele formation [6].

This study sets out to evaluate surgical outcomes in frontal reconstruction, primarily focusing on fracture pattern, surgical approach, and the association of these factors with post-operative complication rates. Our primary objective was to identify injury variables associated with adverse surgical outcomes in frontal trauma patients. The secondary objective was to assess the influence of surgical variables on post-operative complications. A clearer understanding of these associations will allow clinicians to provide tailored, evidence-based interventions to improve surgical planning and outcomes in this complex cohort.

## 2. Materials and Methods

This was an 8-year retrospective cross-sectional study that identified a cohort of 137 patients who sustained frontal sinus and frontal bone fractures between the years 2015 and 2022 at a level one major trauma centre in Central London. These cases were identified from past operating lists and were performed or supervised by dedicated trauma sub-specialist consultants for oral and maxillofacial reconstruction and neurosurgeons. Inclusion criteria consisted of primary reconstruction of frontal sinus fractures, adequate clinical documentation, pre- and post-operative assessment, and known outcomes.

The electronic patient record (EPR) was analysed to assess the following: patient demographics, injury parameters, surgical technique, and complications. Injury parameters included the Lofgren classification (1–4), which accounts for the anterior and posterior tables’ degree of fracture displacement, comminution, and duct FNOD involvement (please see Table 1)

Manson’s classification was then used to analyse the frontobasal fractures, which identified three categories: Type I (isolated cranial base fractures), Type II: cranial base fractures with extension into cranial vault, Type III: cranial base fractures with comminution of lateral and frontal vaults or orbital roof. Other injury parameters were pre-operative pneumocephalus, pre-operative CSF leakage, and cranial nerve injury. Concomitant midface injury data were also recorded, specifically Le Fort-pattern injuries and Naso-orbital-ethmoidal (NOE) complex injuries, and whether these injuries were surgically treated.

The surgical technique data collected included the type of incision, the surgical procedure, and neurosurgical involvement. Acute major complications were described as those requiring return to theatre within six months for cerebrospinal fluid leakage, abscess formation, sinusitis, meningitis, mucocele, or ongoing pneumocephalus [7]. Minor complications included simple wound drainage, infections, or CSF leakage that did not require surgery, as well as delayed cosmetic procedures.

As per local service agreements, there was a strict joint protocol between OMFS and Neurosurgery for frontal sinus obliteration. For more severe injury patterns that required cranialisation, a joint OMFS–Neurosurgical approach was used, with the obliteration performed using pedicle pericranium. With an intact cranial base and FNOD obstruction, OMFS performed sinus obliteration independently, again with a pericranium flap.

Once the cases were identified, the injury parameters were analysed using pre-operative CT, which used the radiography software Sectra PACS IDS7 (Sectra Ltd., Stevenage, UK). In cases that had pre-operative imaging performed at another trust prior to transfer to tertiary care, imaging was requested and securely transferred via the image exchange portal. Injury parameters, specifically anterior and posterior table classification, FNOD obstruction, frontobasal fracture, and pneumocephalus, were analysed using the 3D plane imaging function. Post-operative CT imaging was used to analyse post-operative pneumocephalus.

Statistical analysis was conducted using JASP 0.19.1. Pearson’s chi-square test was used to compare categorical variables/nominal data. The normality of continuous variables (Age and TTS) was assessed using inspections of histograms, Q-Q plots, and Shapiro–Wilk testing. Normality was rejected accordingly. A Mann–Whitney U test was used to compare continuous variables between binary groups. Logistic regression was performed to determine the effects of fracture severity on the likelihood of certain injury outcomes occurring. Exp(B) odds ratios (OR) with 95% exponentiated confidence intervals were calculated to demonstrate the increased odds of adverse outcomes. Model fit was assessed using Nagelkerke R^2^. A two-tailed *p* < 0.05 was considered statistically significant.

## 3. Results

### 3.1. Demographics

**Figure 1:**
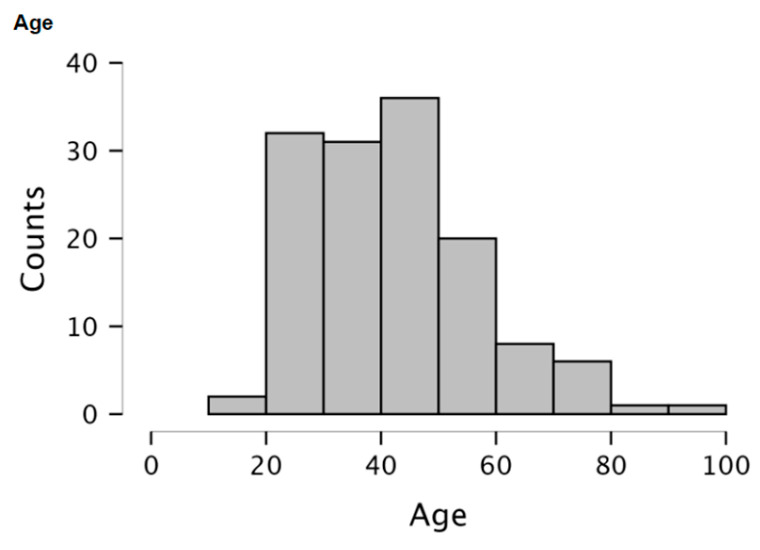
Bar chart representing the distribution of ages within the patient group.

Data were collected on 137 patients with fractures to the frontal vault. Trauma to the upper third of the face predominantly affects the male population, who made up 87.6% of this subset. The average age was 42.88 +/− 14.94 (please see Figure 1) The primary mechanisms of injury were RTC (38.6%), fall (29.1%), and interpersonal violence (24.8%). Age, gender, and patient demographics had no statistically significant association with patient outcome. Gender had no statistically significant association with patient outcome.

### 3.2. Fracture Classification

**Figure 2:**
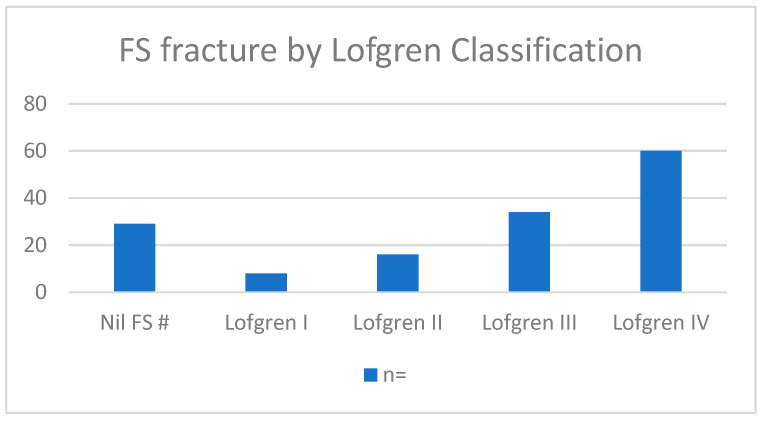
Demonstrates the number of patients within each Lofgren category.

Lofgren Classification (Figure 2).

**Figure 3:**
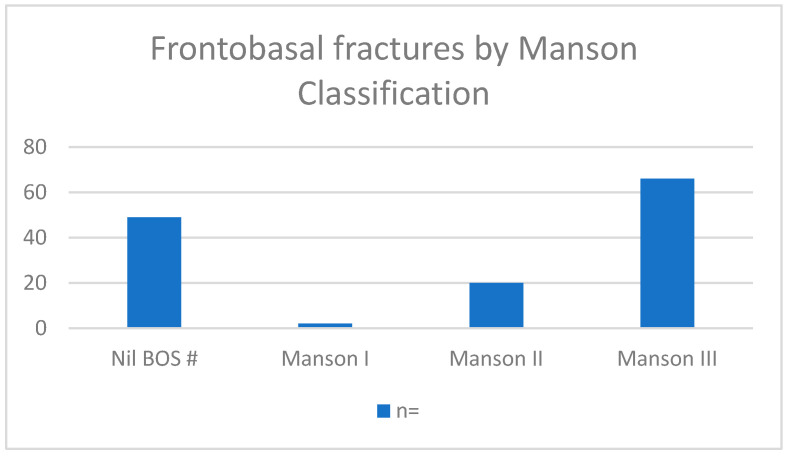
Demonstrates the number of patients in each Manson classification.

Manson Classification (Figure 3).

### 3.3. CSF Leaks

Binary logistic regression demonstrated a significant association between Lofgren classification and post-operative dural injury/CSF leak (χ^2^(1) = 7.67, *p* = 0.006). For each one-point increase in Lofgren classification, the odds of dural injury increased by approximately seven-fold (OR = 7.112, 95% CI [0.979, 51.642]). The same significance was not seen when assessing the Manson classification (χ^2^(1) = 2.227, *p* = 0.136).

There was a statistically significant association between a longer TTS and a higher incidence of CSF (*p* = 0.041). Frontobasal fracture (*p* = 0.032), nasofrontal tract involvement (*p* = 0.015), and pre-op pneumocephalus (*p* = 0.005) were all predictive of CSF leak. Cranialisation cases (*p* < 0.001) were associated with CSF leak. Coronal approach and obliteration were not associated with CSF leak outcome. Midface injury (NOE or Le Fort pattern), including those that were surgically managed, did not affect complication rates. Please see Table 2 for the complete breakdown of the CSF leak statistics.

### 3.4. Post-Op Infection

Increased time to surgery (TTS) appeared to be related to a higher incidence of post-op infection (*p* = 0.050). Binary logistic regression demonstrated a significant association between Lofgren classification and post-operative infection (χ^2^(1) = 4.540, *p* = 0.033). For each one-point increase in the Lofgren classification, the odds of post-op infection increased by approximately three-and-a-half-fold (OR = 3.485, 95% CI [0.851, 14.277]). The same significance was not seen when assessing the Manson classification (χ^2^(1) = 2.915, *p* = 0.088).

FNOD involvement (*p* = 0.016) was predictive of post-op infection, but frontobasal fracture and pneumocephalus were not. Cranialisation cases (*p* = 0.016) were more likely to be associated with post-op infection. Coronal approach and obliteration were not associated.

Midface injuries (NOE or Le Fort pattern), including those that were surgically managed, did not affect complication rates.

Please see Table 3 for the complete breakdown of the post-op infection statistics.

### 3.5. RTT

TTS had no statistically significant association with RTT. Binary logistic regression demonstrated a significant association between Lofgren classification and return to theatre (χ^2^(1) = 7.669, *p* = 0.006). For each one-point increase in the Lofgren classification, the odds of returning to theatre increased by approximately seven-fold (OR = 7.112, 95% CI [0.979, 51.642]). The same significance was not seen when assessing the Manson classification (χ^2^(1) = 2.258, *p* = 0.133). Frontobasal fracture (*p* = 0.02) and pre-op pneumocephalus (*p* = 0.005) were both predictive of RTT, but nasofrontal tract injurywas not. Cranialisation (*p* < 0.001) cases were more likely to be associated with RTT. Coronal approach and obliteration were not associated.

Midface injuries (NOE or Le Fort pattern), including those that were surgically managed, did not affect complication rates.

Please see Table 4 for the complete breakdown of RTT statistics.

### 3.6. Surgical Approach and Complications

Of the 137 patients in this cohort, 91 were surgically managed, and the remaining 46 were managed conservatively. A total of 37.4% (*n* = 34) of patients had a simple ORIF of the anterior table. In total, 47.3% (*n* = 43) of patients had cranialisation and 15.4% (*n* = 14) had obliteration. The average time to surgery (TTS) was 7.09 days +/− 7.70.

The major surgical complication rate requiring return to theatre was 13.0% (*n* = 12), which reflects that in the existing literature [14]. Of the 12 patients that required a return to theatre, 5.4% (*n* = 5) did so for CSF leaks, 5.4% (*n* = 5) for infection, and 2.2% (*n* = 2) for haematoma/bleeding.

A further four patients had post-operative cosmetic procedures performed that were related to their upper third facial injuries: these included endoscopic brow-lifting and lipofiller for cosmetic defects. These were deemed minor complications and predictable sequelae to major craniomaxillofacial trauma.

Overall, 12% (*n* = 11) of patients had post-operative CSF leaks. Of these, 4.3% (*n* = 4) had spontaneous resolution, 2.1% (*n* = 2) required a lumbar drain, 3.2% (*n* = 3) had endoscopic repair, and 2.1% (*n* = 2) had an open repair, which required cranialisation to be performed a second time.

Of the 11 patients with post-operative infection complications, 6 of these patients developed intracranial abscess or infected haematoma necessitating surgical management. These patients had surgical drainage via the previous surgical site, including two patients who required cranialisation to be performed a second time. The remaining five were managed conservatively with broad-spectrum antibiotics. Table 5 shows the interval for each RTT patient per indication.

## 4. Discussion

Fractures to the FS carry high morbidity due to the frequent involvement of both the anterior and posterior tables, FNOD, the cranial base, and intracranial involvement, resulting in pneumocephalus, CSF leakage, and cerebral injury. We have examined a comprehensive range of surgical factors that may predict outcomes for patients. Overall, the major surgical complication rate was 13% (*n* = 12) in our patient cohort, which reflects that in the existing literature [14]. Increasing injury complexity, represented by the Lofgren classification, was identified as a risk factor for RTT—for every one-point increase in category, a seven-fold risk increase was seen. In total, 11 of the 12 RTT patients were category 4 in the Lofgren classification (which is specified as “posterior table fracture with significant displacement or comminution, intracranial injury or CSF leak”).

**Figure 4:**
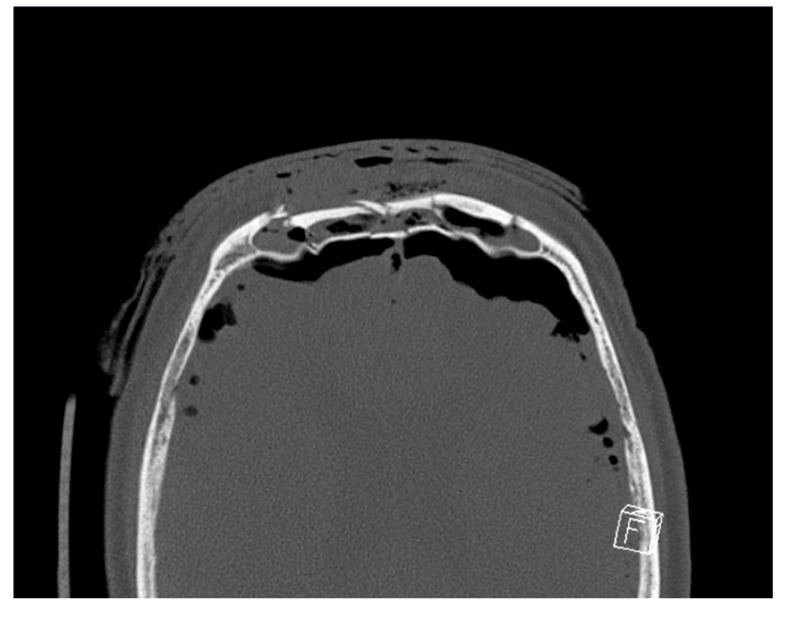
Axial CT section of a patient showing comminuted outer and inner tables and large volume pneumocephalus.

We can propose that the significant posterior wall disruption and FNOD involvement represented by Lofgren categories 3 and 4 allow for the ingress of microorganisms from the external environment into the intracranial cavity, thus increasing the risk of infection. A higher Lofgren classification was therefore also a risk factor for post-operative infection, with each one-point increase in category corresponding to a 3.5-fold increase in the odds ratio. Figure 4 shows an example of a CT which was categorised as Lofgren 4.

Increasing Lofgren classification was also associated with CSF leakage, with each one-point increase in category corresponding to a seven-fold increase in the risk of CSF leakage. Dalla Torre et al. saw a high incidence of CSF leakage in patients who had fractures of greater than 5 mm [15]. With a breach of the posterior wall, pneumocephalus may occur [16], which was also associated with increased RTT in our dataset.

FNOD involvement was seen to be predictive of both CSF leakage and infection. The literature concludes that FNOD disruption occurs in most cases with anterior and posterior table FS involvement, which, as discussed, provides a route for communication between the external environment and the intracranial space [17]. Despite this, FNOD was not associated with overall RTT.

Manson et al. [12] found that Type III fractures carried the highest complication risk (25%). Our data does not show a significant association between Manson classification and RTT; however, frontobasal fracture as a binary datapoint was predictive of complications. While the statistical analysis regarding Manson classification and adverse surgical outcomes generated in this study failed to reach significance, the unequal division of cases between Manson categories may have acted as confounders, and a greater sample size could highlight a true association.

Increased time to surgery (TTS) was predictive of post-op CSF leak and post-op infection. When examining our surrogate variables (Lofgren and Manson) against TTS, they did not reveal any significant association; therefore, our results demonstrate that TTS is an important factor, regardless of injury severity. However, it must be noted that overall injury burden may delay treatment in these cases, explaining some of the association.

Cranialisation was significantly associated with post-op CSF leak, infection, and RTT. This is expected, given that the indication for this procedure entails the involvement of the intracranial space, severe disruption of the posterior table, and FNOD involvement necessitating the involvement of neurosurgery. This is most likely unavoidable. Interestingly, sinus obliteration was not predictive of RTT, CSF leak, or infection. This may be because the most common complication of sinus obliteration is mucocele development, which occurs as a late complication, ranging from 1 to 40 years post-operatively (with an average of 7.5 years) [18].

## 5. Conclusions

Injuries to the FS frequently involve the anterior and posterior tables, FNOD, the cranial base, and the intracranial space. The consequences of these injuries have high morbidity, resulting in pneumocephalus, CSF leakage, infection, and cerebral injury. We have examined a comprehensive range of injury-related and operative surgical variables that may predict outcomes for patients. The significant increase in the complication risk for higher Lofgren categories, as exemplified by the odds ratio analysis, highlights the importance of both posterior wall and FNOD involvement in FS complex injury profiles. A 7-fold increase in risk for CSF leakage and RTT (and 3.5 for infection) for each Lofgren category may provide invaluable data for the surgeon when making decisions about indications for treatment and surgical approach. While the statistical analysis regarding Manson classification and adverse surgical outcomes generated in this study failed to reach significance, the unequal division of cases between Manson categories may have acted as confounders, and a greater sample size could highlight a true association. Cranialisation procedures were also predictive of post-operative complications. Understanding risk profiles in the management of FS fractures is vital in order to help clinicians mitigate these risks and also to educate patients better, especially during the consent process. Further research could look at the medical and social risk factors that increase complication rates in this patient cohort.

## Figures and Tables

**Table 1:** Lofgren Classification of Frontal Sinus Injuries.

Lofgren Classification	Category
1	Minimally displaced anterior table fracture (<1–2 mm) without nasofrontal recess injury
2	Fractures of the anterior table (>2 mm) without the involvement of the nasofrontal recess or in patients with an obvious cosmetic forehead deformity
3	Comminuted anterior table fractures with a linear nondisplaced posterior table fracture or involvement with the frontonasal duct. Significant mucosal disruption of the sinus or a severely comminuted fracture of the anterior table
4	Posterior table fractures with significant displacement or comminution, intracranial injury, or CSF leak

**Table 2:** Demographics, mechanism of injury, time to surgery, fracture pattern, injury severity, surgical approach, and their respective associations with CSF leakage. Statistical significance is presented as a *p*-value. Data are presented as the number per group.

		Dural Injury/CSF Leak (*n* = )	No Dural Injury (*n* = )	Test Statistic and *p*-Value
Age				(U = 458.5, *p* = 0.934)
Gender	Male	11	69	(χ^2^(1) = 2.027, *p* = 0.154)
	Female	0	13	
Mechanism	RTC	4	29	(χ^2^(1) = *p* = 0.880)
	IPV	2	19	
	Fall	4	28	
	Sport	1	3	
	Other	0	3	
TTS				(U = 280.5, *p* = 0.041)
Lofgren	1	0	1	(χ^2^(1) = 7.67, *p* = 0.006)
	2	0	11	
	3	1	25	
	4	10	40	
Manson	1	0	0	(χ^2^(1) = 2.227, *p* = 0.136)
	2	0	6	
	3	11	51	
Frontobasal fracture	Y	11	57	
	N	0	25	(χ^2^(1) = 4.587, *p* = 0.032)
Nasofrontal tract	Y	11	30	
	N	0	52	(χ^2^(1) = 5.941, *p* = 0.015)
Le Fort Injury	Y	3	25	
	N	8	57	(χ^2^(1) = 0.048, *p* = 0.827)
NOE injury	Y	3	17	
	N	8	65	(χ^2^(1) = 0.246, *p* = 0.620)
Pre-op pneumocephalus	Y	11	46	(χ^2^(1) = 7.879, *p* = 0.005)
	N	0	36	
Obliteration	Y	0	14	(χ^2^(1) = 2.265, *p* = 0.132)
	N	9	54	
Cranialisation	Y	11	32	(χ^2^(1) = 13.427, *p* = <0.001)
	N	0	46	(χ^2^(1) = 13.427, *p* = <0.001)
Midface fracture also treated	Y	5	27	(χ^2^(1) = 0.674, *p* = 0.412)
	N	6	55	

**Table 3:** Demographics, mechanism of injury, time to surgery, fracture pattern, injury severity, surgical approach, and their respective associations with post-operative infection. Statistical significance is presented as a *p*-value. Data are presented as the number per group.

		Post-Op Infection (*n* = )	No Post-Op Infection (*n* = )	Test Statistic and *p*-Value
Age				(U = 570.0, *p* = 0.115)
Gender	Male	11	67	(χ2(1) = 2.085, *p* = 0.149)
	Female	0	13	
Mechanism	RTC	2	31	(χ2(1) = 2.085, *p* = 0.149)
	IPV	4	16	
	Fall	4	27	
	Sport	1	3	
	Other	0	3	
TTS				(U = 280, *p* = 0.050)
Lofgren	1	0	1	(χ2(1) = 4.540, *p* = 0.033)
	2	0	11	
	3	2	24	
	4	9	41	
Manson	1	0	2	(χ2(1) = 2.915, *p* = 0.088)
	2	2	2	
	3	8	54	
Frontobasal fracture	Y	10	56	(χ2(1) = 2.122, *p* = 0.145)
	N	1	24	
Nasofrontal tract	Y	11	51	
	N	0	29	(χ2(1) = 5.853, *p* = 0.016)
Le fort injury	Y	5	21	(χ2(1) = 1.748, *p* = 0.186)
	N	6	59	
NOE injury	Y	4	15	
	N	7	65	(χ2(1) = 1.816, *p* = 0.178)
Pre-op pneumocephalus	Y	9	49	(χ2(1) = 1.770, *p* = 0.183)
	N	2	31	
Obliteration	Y	1	13	(χ2(1) = 0.323, *p* = 0.570)
	N	8	56	
Cranialisation	Y	9	34	(χ2(1) = 5.820, *p* = 0.016)
	N	2	45	
Midface fracture also treated	Y	6	25	
	N	5	55	(χ2(1) = 2.336, *p* = 0.126)

**Table 4:** Demographics, mechanism of injury, time to surgery, fracture pattern, injury severity, surgical approach, and their respective associations with RTT. Statistical significance is presented as a *p*-value. Data are presented as the number per group.

		RTT (*n* = )	No RTT (*n* = )	Test Statistic and *p*-Value
Age				(U = 568.0, *p* = 0.272)
Gender	Male	12	66	(χ2(1) = 2.304, *p* = 0.129)
	Female	0	13	
Mechanism	RTC	3	30	(χ2(1) = 4.085, *p* = 0.043)
	IPV	3	17	
	Fall	5	26	
	Sport	1	3	
	Other	0	3	
TTS				(U = 324.5, *p* = 0.077)
Lofgren	1	0	1	(χ2(1) = 7.669, *p* = 0.006)
	2	0	11	
	3	1	25	
	4	11	40	
Manson	1	0	0	(χ2(1) = 2.258, *p* = 0.133)
	2	2	2	
	3	10	52	
Frontobasal fracture	Y	12	54	(χ2(1) = 5.236, *p* = 0.022)
	N	0	25	
Nasofrontal tract	Y	11	51	
	N	1	28	(χ2(1) = 3.526, *p* = 0.050)
Le fort injury	Y	3	25	
	N	8	57	(χ2(1) = 0.048, *p* = 0.827)
NOE injury	Y	2	17	
	N	10	62	(χ2(1) = 0.148, *p* = 0.700)
Pre-op pneumocephalus	Y	12	46	(χ2(1) = 7.865, *p* = 0.005)
	N	0	33	
Obliteration	Y	0	14	(χ2(1) = 2.801, *p* = 0.094)
	N	11	53	
Cranialisation	Y	11	32	(χ2(1) = 10.689, *p* = 0.001)
	N	1	46	
Midface fracture also treated	N	7	53	(χ2(1) = 0.356, *p* = 0.551)
	Y	5	26	

**Table 5:** RTT indication and intervals.

Indication	RTT Interval (Days)
Bleeding	0
Haematoma	1
Infection	6
Infection	6
Infection	14
CSF leak	21
CSF leak	31
Infection	34
Infection	51
CSF leak	86
CSF leak	307
CSF leak	492

## Data Availability

The raw data supporting the conclusions of this study are available from the authors upon reasonable request.

## References

[1] Rohrich R.J., Hollier L.H. (1992). Management of Frontal Sinus Fractures: Changing Concepts. Clin. Plast. Surg..

[2] Vu A.T., Patel P.A., Chen W., Wilkening M.W., Gordon C.B. (2015). Pediatric frontal sinus fractures: Outcomes and treatment algorithm. J. Craniofac. Surg..

[3] Arnold M.A., Tatum S.A. (2019). Frontal Sinus Fractures: Evolving Clinical Considerations and Surgical Approaches. Craniomaxillofac. Trauma Reconstr..

[4] Strong E.B., Pahlavan N., Saito D. (2006). Frontal sinus fractures: A 28-year retrospective review. Otolaryngol.–Head Neck Surg..

[5] Fox P.M., Garza R., Dusch M., Hwang P.H., Girod S. (2014). Management of frontal sinus fractures: Treatment modality changes at a level I trauma center. J. Craniofac. Surg..

[6] Jing X.L., Luce E. (2019). Frontal Sinus Fractures: Management and Complications. Craniomaxillofac. Trauma Reconstr..

[7] Peng X.L., Yue Z.Z., Zhang Y.L., Sun P.Y. (2023). Nasal Sinus Mucoceles Manifesting Ocular Symptoms. J. Craniofacial Surg..

[8] Nahum A.M. (1975). The Biomechanics of Maxillofacial Trauma. Clin. Plast. Surg..

[9] Schultz R.C. (1970). Supraorbital and glabellar fractures. Plast. Reconstr. Surg..

[10] Nelson E.L., Melton L.J., Annegers J.F. (1984). Incidence of skull fractures in Olmsted County, Minnesota. Neurosurgery.

[11] Ratilal B.O., Costa J., Sampaio C. (2006). Antibiotic prophylaxis for preventing meningitis in patients with basilar skull fractures. Cochrane Database Syst. Rev..

[12] Manson P.N., Stanwix M.G., Yaremchuk M.J., Nam A.J., Hui-Chou H., Rodriguez E.D. (2009). Frontobasal fractures: Anatomical classification and clinical significance. Plast. Reconstr. Surg..

[13] Lee Y., Choi H.G., Shin D.H., Uhm K.I., Kim S.H., Kim C.K., Jo D.I. (2014). Subbrow approach as a minimally invasive reduction technique in the management of frontal sinus fractures. Arch. Plast. Surg..

[14] Al-Moraissi E.A., Alyahya A., Ellis E. (2021). Treatment of Frontal Sinus Fractures: A Systematic Review and Meta-analysis. J. Oral Maxillofac. Surg..

[15] Dalla Torre D., Burtscher D., Kloss-Brandstätter A., Rasse M., Kloss F. (2014). Management of frontal sinus fractures—Treatment decision based on metric dislocation extent. J. Cranio-Maxillofac. Surg..

[16] Das J.M., Munakomi S., Bajaj J. Pneumocephalus. Critical Findings in Neuroradiology. https://www.ncbi.nlm.nih.gov/books/NBK535412/.

[17] Metzinger S.E., Metzinger R.C. (2009). Complications of Frontal Sinus Fractures. Craniomaxillofac. Trauma Reconstr..

[18] Javer A.R., Alandejani T. (2010). Prevention and Management of Complications in Frontal Sinus Surgery. Otolaryngol. Clin. N. Am..

